# Machine learning-based mortality prediction for pediatric fulminant myocarditis using cytokine profiles

**DOI:** 10.1038/s41598-026-50260-4

**Published:** 2026-04-24

**Authors:** Sihuan Jing, Takanori Suzuki, Kenta T. Suzuki, Yoji Nomura, Katsuyuki Kunida, Yuichi Sakumura, Hidetoshi Uchida, Kazuyoshi Saito, Ryoichi Ito, Machiko Kito, Satoru Kawai, Alejandro A. Floh, Aamir Jeewa, Junichiro Yoshimoto, Tetsushi Yoshikawa, Kazushi Yasuda

**Affiliations:** 1 Graduate School of Science and Technology, Nara Institute of Science and Technology, Nara, Japan; 2 Department of Pediatrics, Fujita Health University, Aichi, Japan; 3 Labatt Family Heart Centre, The Hospital for Sick Children, Toronto, ON Canada; 4 Data Science Center, Nara Institute of Science and Technology, Ikoma, Nara Japan; 5 Department of Pediatric Cardiology, Aichi Children’s Health and Medical Center, Aichi, Japan; 6 Department of Computational Biology, Fujita Health University, Aichi, Japan; 7 Division of Computational Science, International Center for Brain Science, Fujita Health University, Aichi, Japan; 8 Cardiac Critical Care, Department of Critical Care Medicine, Hospital for Sick Children, University of Toronto, Toronto, ON Canada; 9 Division of Pediatric Cardiology, Labatt Family Heart Centre, University of Toronto, Toronto, Canada; 10 Department of Biomedical Data Science, Fujita Health University, Aichi, Japan

**Keywords:** Fulminant myocarditis, Cytokine, Machine learning analysis, Mortality prediction, Feature importance analysis, Biomarkers, Cardiology, Diseases, Immunology, Medical research

## Abstract

Fulminant myocarditis (FM) is a rare but life-threatening pediatric condition that can rapidly progress to cardiogenic shock and fatal arrhythmia. Prognostic biomarkers in FM are essential for optimizing treatment strategies. Although inflammatory cytokines have been associated with the pathogenesis of FM, their prognostic value remains unclear. This study aimed to identify mortality-associated markers by integrating cytokine profiles and clinical variables through a machine learning approach. We retrospectively analyzed 21 pediatric FM cases from two tertiary centers (2012–2022). At admission, 37 cytokines and 14 clinical parameters were assessed. Partial least squares discriminant analysis was employed to identify prognostic features, with variable importance in projection scores quantifying their contribution. Model performance was evaluated using repeated stratified 3-fold cross-validation with 100 repeats. For each repeat, predictions obtained from the three folds were averaged at the sample level, and performance metrics were computed from these fold-averaged predictions. Statistical significance was determined via the Benjamini–Hochberg method at a false discovery rate of 0.05. Of the 51 features analyzed, 18 emerged as key predictors, 15 cytokines and 3 clinical parameters, with variable importance in projection (VIP) scores above 1.0. Seven cytokines (TNF-α, MIP-1α, M-CSF, IL-8, IL-6, IL-15, and IP-10) were both statistically significant and highly important. TNF-α had the highest VIP score among all predictors. Three clinical parameters (CK-MB, pH, and lactate) were also linked to poor outcomes. The model performed robustly, with an area under the receiver operating characteristic curve (AUROC) of 0.912, an area under the precision–recall curve (AUPRC) of 0.874, 83.8% accuracy, 71.0% sensitivity, and 90.1% specificity. Furthermore, the cytokine-enriched model showed higher predictive performance than the clinical-only model across accuracy, AUROC, and AUPRC. Analysis of cytokine profiles using machine learning may identify biomarkers associated with mortality risk in pediatric FM. TNF-α emerged as a key cytokine associated with mortality, supporting its potential role as a prognostic biomarker. Importantly, cytokine markers added prognostic value beyond routine clinical variables.

**Supplementary Information:**

The online version contains supplementary material available at 10.1038/s41598-026-50260-4.

## Introduction

Fulminant myocarditis (FM) is characterized by extensive and severe myocardial inflammation with the potential for developing malignant arrhythmias and cardiogenic shock. Although advances in critical care management and mechanical circulatory support (MCS) have improved outcomes, FM remains associated with a high risk of mortality. This is largely due to challenges in establishing the diagnosis and suboptimal timing of medical therapy and extracorporeal membrane oxygenation (ECMO). These issues highlight the importance of early recognition and treatment^1,2^. Previous studies showed that elevated B-type natriuretic peptide (BNP) levels, reduced left ventricular ejection fraction (LVEF), and acidosis were predictors of poor prognosis in FM^3,4^. However, in one study, only 8 out of 28 patients (29%) received MCS, reflecting its limited use and potentially restricting the applicability of the findings^4^. These studies also had several limitations, including their single-center and the absence of biopsy-confirmed myocarditis, and reliance solely on univariate analysis.

Cytokines play a critical role in the pathophysiology of myocarditis, with tumor necrosis factor-alpha (TNF-α) and IL-6 recognized as established markers of disease severity^5,6^. In our previous study, we used machine learning to show that multiple cytokines can stratify the severity of acute myocarditis (AM) and its more severe form, FM in children at the time of admission^7^. However, prognostic factors specifically associated with FM remain unclear. This study aims to identify predictors of poor prognosis in pediatric FM, a rare but highly severe condition through the integration of clinical characteristics and cytokine profiling.

Medical applications of machine learning have advanced the analysis of high-dimensional clinical datasets^8,9^. These methods can uncover latent associations, enable robust classification, and improve predictive precision in clinical research by modeling complex relationships among correlated variables^10^. Previously, partial least squares discriminant analysis (PLS-DA), a supervised dimensionality reduction method that projects predictors onto latent components to maximize class separation, and variable importance in projection (VIP) scores, which quantify the contribution of each variable to the PLS-DA model, were employed for feature selection; this approach identified novel plasma biomarkers that may help elucidate disease mechanisms, improve prognostic accuracy, and enable personalized treatment^8,11^. By integrating cytokine profiling with machine learning, this study seeks to improve elucidate the inflammatory landscape of FM, and identify key biomarkers for potential therapeutic targeting.

## Materials and methods

### Patients

This study was conducted using a retrospective design. Patients with FM admitted to Aichi Children’s Health and Medical Center and Fujita Health University between January 2012 and December 2022 were retrospectively analyzed. AM was defined by at least one of the following criteria within 30 days of symptom onset: (1) histopathological evidence of inflammatory cell infiltration and myocardial injury on biopsy or (2) clinical presentation consistent with heart failure, characterized by a progressive course and elevated serum high-sensitivity troponin or creatine kinase MB (CK-MB) levels^2^. The cutoff levels used for diagnosis were 0.014 ng/mL for high-sensitivity troponin and 13 U/L for CK-MB, based on our institutional reference ranges. Among patients with AM, cases exhibiting low cardiac output syndrome requiring MCS owing to cardiogenic shock, life-threatening ventricular tachycardia (VT), or bradyarrhythmia such as complete atrioventricular block (CAVB) were classified as FM^1,12^. Of the 21 patients included, 12 (57.1%) were diagnosed by endomyocardial biopsy, while the remaining cases were diagnosed clinically. In the acute phase treatment of these patients, steroids were administered in 14 cases, and intravenous immunoglobulin was used in 18 cases. This study received approval from the institutional review boards (IRB) of Aichi Children’s Health and Medical Center and Fujita Health University (Approval No. 2019027 and HM21-575). Informed consent was obtained through an opt-out process in accordance with institutional guidelines, ensuring appropriate disclosure to all patients or guardians.

### Clinical data

The clinical data assessed in this study included sex, age, height, body weight, body surface area, arterial blood gas pH, lactate, BNP, troponin T, CK-MB, and LVEF. The interval from symptom onset to hospital admission (days) was also documented, along with the presence of VT and CAVB. The primary endpoint was in-hospital mortality.

### Cytokine profile

Whole blood was collected in anticoagulant-free tubes, and serum was separated by centrifugation and stored at − 80 °C until analysis. Cytokine measurements were performed once, using the first blood sample obtained immediately after pediatric intensive care unit admission. A total of 37 cytokines were measured using the same methodology described in our previous study^7^. The cytokines analyzed in this study are listed in Supplementary Table 1. Cytokines were quantified using the MILLIPLEX^®^ Human Cytokine/Chemokine/Growth Factor Panel A Magnetic Bead Panel (Merck KGaA, Darmstadt, Germany; Cat. HCYTA-60 K-PX38). Concentrations were reported in pg/mL, and values below the lower limit of detection (LOD) were replaced with LOD/2. All samples underwent one freeze–thaw cycle, and hemolysis was assessed visually; no hemolyzed samples were included.

A set of seven calibration standards was prepared by five-fold serial dilution of the reference standard provided in the kit, and each plate included two internal quality-control samples. Fluorescent signals were acquired using a Luminex 200 system with XPONENT software (Luminex Corporation, Austin, TX, USA) and quantified using Milliplex Analyst 5.0 (Merck KGaA, Darmstadt, Germany). Standard curves demonstrated excellent linearity (*R* > 0.99 for all analytes). All measurements were performed on a single assay plate, and therefore, no inter-plate batch effects were present.

### Statistical analysis

Categorical variables were evaluated using Fisher’s exact test. For clinical characteristics and cytokine data that deviated from normal distribution, the non-parametric Mann–Whitney U test was employed. To account for multiple cytokine comparisons, *p*-values were adjusted using the Benjamini–Hochberg procedure, with a false discovery rate (FDR)-adjusted q-value of ≤ 0.05 considered statistically significant.

### Data preprocessing

To ensure comparability across variables with differing scales, the data analysis process began with preprocessing to standardize all features. Specifically, z-score normalization^13^ was used to transform each feature to have a mean of 0 and a standard deviation of 1, ensuring that all variables contributed equally to the model. This process is represented as follows:

1 $$\:\begin{array}{c}z=\frac{x-\mu\:}{\sigma\:}\end{array}$$

where $\:z$ is the standardized value, $\:x$ is the original value, $\:\mu\:$ is the mean of the feature, and $\:\sigma\:$ is the standard deviation of the feature.

### Hierarchical clustering

Hierarchical clustering was used to group similar data elements into clusters and visualize them. The clustering results were represented using a dendrogram, a two-dimensional tree-like diagram depicting nested clusters^14^. Ward’s linkage method was applied^15^, which minimizes within-cluster variance, and Euclidean distance was used as the dissimilarity metric between variables and patients. The normalized data from preprocessing served as input for the clustering process. In this study, hierarchical cluster analysis and heatmap generation were performed to investigate relationships among clinical parameters, cytokine profiles, and patient survival outcomes. In the heatmap, rows represented measured parameters (clinical characteristics and cytokines), while columns corresponded to individual patients, color-coded by outcome (green for survival and orange for mortality). Hierarchical clustering was applied to organize the rows based on expression pattern similarity across patients. The dendrogram on the left side of the heatmap depicted the clustering hierarchy, offering insights into parameters with shared expression patterns and potential biological relevance.

### Machine learning method

#### PLS-DA

PLS-DA was used to reduce the dataset’s high dimensionality and classify patients into survival and mortality groups. This technique is well-suited for datasets with correlated variables, as it projects the data onto latent variables that capture variance and outcome-relevant patterns. In doing so, PLS-DA reduces dimensionality while identifying features most critical for differentiating patient outcomes. This makes it particularly valuable for pinpointing key cytokines and clinical parameters linked to prognosis.

#### Model evaluation

To validate the classification model, repeated stratified k-fold cross-validation was employed. This approach preserves the class proportions in each fold while providing a robust and unbiased estimate of model performance. To prevent overfitting and data leakage, all analytical procedures, including data preprocessing (z-score normalization), feature selection based on VIP scores, and model construction, were performed strictly within each cross-validation fold. Specifically, stratified 3-fold cross-validation was repeated 100 times. In each repetition, the model was trained on two folds and evaluated on the remaining fold, and this process was iterated so that each fold served once as the validation set. The area under the receiver operating characteristic curve (AUROC) and the area under the precision–recall curve (AUPRC) were calculated for each of the three folds, and the mean values across the folds were used as the performance metrics for that repetition. Furthermore, receiver operating characteristic (ROC) curves and precision–recall (PR) curves were generated for each repetition to visually assess classification performance across different decision thresholds. To quantify the uncertainty and stability of the model performance, the standard deviations of AUROC and AUPRC across the 100 repetitions were calculated. This repeated cross-validation framework enables reliable assessment of predictive performance while accounting for sampling variability in small and potentially imbalanced datasets. In addition to AUROC and AUPRC, the model was further evaluated using conventional machine learning performance metrics, including accuracy, precision, recall (sensitivity), specificity, and F1 score. These metrics were defined using true positives (TP), false positives (FP), true negatives (TN), and false negatives (FN) as follows:

2 $$\:Accuracy=\frac{TP+TN}{TP+TN+FP+FN}$$

3 $$\:Precision=\frac{TP}{TP+FP}$$

4 $$\:Recall\:\left(Sensitivity\right)=\frac{TP}{TP+FN}$$

5 $$\:Specificity=\frac{TN}{TN+FP}$$

6 $$\:{F}_{1}\:score=2\cdot\:\frac{\mathrm{Precision}\cdot\:\mathrm{Recall}}{\mathrm{Precision}+\mathrm{Recall}}$$

#### Feature importance analysis

The VIP score was used to identify the most relevant cytokines for distinguishing between survival and mortality outcomes. The VIP score quantifies the contribution of each variable in the PLS-DA model also applied in this study. Importantly, the VIP score reflects the contribution of each variable within the latent component structure of the model and does not represent an independent measure of association. These scores are derived from the weighted sum of squared correlations between cytokine levels and the PLS-DA components:

$$\:{VIP}_{j}=\sqrt{p\sum\:_{a=1}^{A}\left(\frac{SS\left({T}_{a}\right){w}_{ai}^{2}}{\sum\:_{j=1\:}^{p}{w}_{ai}^{2}}\right)}$$

where $\:{VIP}_{j}$ is the VIP score for feature $\:j$; $\:p$ is the total number of features; $\:A$ is the number of PLS components (latent variables); $\:{w}_{ai}$ is the weight of feature $\:j$ in component a, $\:SS\left({T}_{a}\right)$　represents the variance in the response variable explained by component $\:a;and$ $\:\sum\:_{j=1\:}^{p}{w}_{ai}^{2}\:$is the sum of the squared weights of all features for component $\:a$.

Features with higher VIP scores were considered more important for distinguishing between the survival and mortality groups. Typically, features with VIP scores greater than 1 were regarded as significant contributors to the model^8^. This approach enabled the identification of key cytokines that play a critical role in predicting survival outcomes, providing insight into the inflammatory processes underlying pediatric FM. In this binary classification task, coefficients were extracted from the first latent component (PLS1), which best reflected the separation between survival and mortality groups. This made the interpretation of each cytokine’s role more straightforward and reliable. In addition, VIP scores were computed within each resampling iteration rather than from a single model fitted to the full dataset, to avoid optimistic bias. The VIP scores highlighted cytokines and clinical markers critical for predicting survival, offering mechanistic insight into dysregulated inflammatory pathways in pediatric FM. To assess the stability of the VIP score estimation, 2,000 bootstrap resamplings were performed using the 0.632 + bootstrap method. For each feature, the distribution of VIP scores was summarized using the median and interquartile range (IQR), along with the proportion of resamples with VIP > 1. Moreover, the lower and upper bounds were defined as bias-corrected and accelerated (BCa) bootstrap confidence intervals derived from the empirical VIP distributions. These results are summarized in Supplementary Table 3.

#### Permutation testing

To evaluate whether the observed model performance could be attributed to chance, a permutation test was conducted by randomly shuffling the class labels 1,000 times. For each permutation, the model was retrained, performance was recalculated, and the resulting null distribution was used to assess the statistical significance of the original performance.

All analyses were performed using R (version 4.5.1 R Foundation for Statistical Computing, Vienna, Austria). Model training and evaluation were implemented using the caret package (version 7.0–1: Comprehensive R Archive Network [CRAN], R Foundation for Statistical Computing, Vienna, Austria), PLS-DA modeling was conducted with ropls (version 1.42.0: Comprehensive R Archive Network [CRAN], R Foundation for Statistical Computing, Vienna, Austria), and bootstrap analyses were performed using bootnet (version 1.6: Comprehensive R Archive Network [CRAN], R Foundation for Statistical Computing, Vienna, Austria). This study is reported in accordance with the TRIPOD-ML checklists for reporting machine learning-based prediction models (see Supplementary Table 2).

## Results

### Statistical comparison of clinical characteristics and cytokine profile on survival and mortality groups

**Fig. 1:**
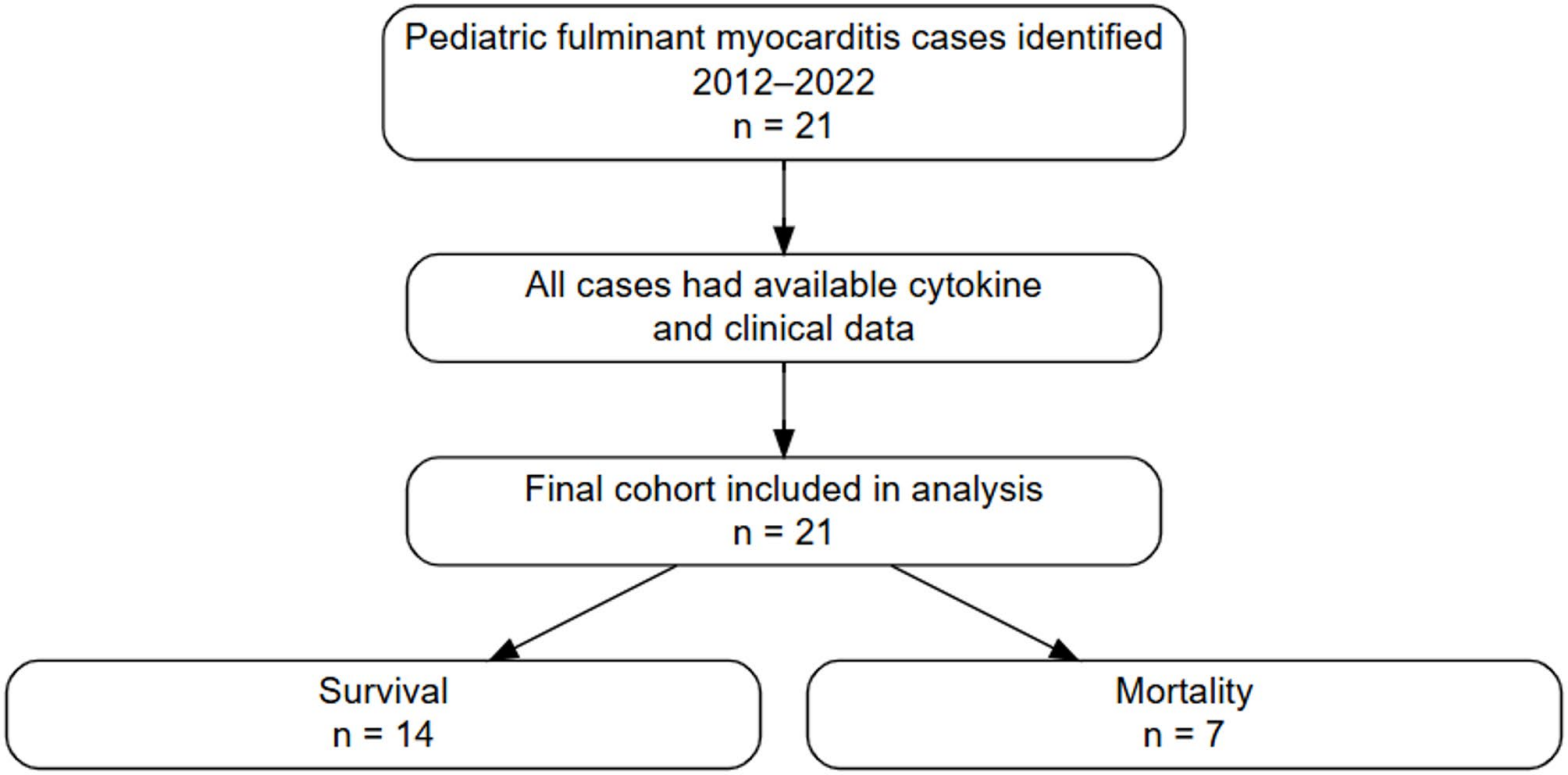
Flow diagram of patient selection and study cohort classification. All consecutive pediatric fulminant myocarditis cases identified during the study period were included in the final analysis.

Baseline demographic characteristics and laboratory test results for the survival (*n* = 14) and mortality (*n* = 7) groups are summarized in Table 1; Fig. 1. The median age was 113 months (range: 2–167 months) in the survival group and 150 months (range: 14–180 months) in the mortality group. The survival group comprised 4 males (28.6%), while the mortality group comprised 3 males (42.9%). The causes of death in the mortality group were as follows: severe neurological dysfunction (*n* = 3), multiple organ failure (*n* = 2), sepsis (*n* = 1), and cardiac deterioration following ECMO weaning (*n* = 1). No statistically significant differences were observed in demographic parameters between the groups. Notably, sex, age, troponin T, and LVEF showed no significant differences despite clinical relevance: male sex (survival: 28.6% vs. mortality: 42.9%, *p* = 0.638), age (survival: 113 months [2–167] vs. mortality: 150 months [14–180], *p* = 0.636), troponin T (survival: 1.96 ng/mL [0.33–9.35] vs. mortality: 2.72 ng/mL [0–24.7], *p* = 0.971), and LVEF (survival: 30.7% [9–72] vs. mortality: 10.7% [8–40], *p* = 0.117). Arterial blood pH was the only clinical parameter that differed significantly between groups. The mortality group exhibited significantly lower pH than that of the survival group (mortality: 7.02 [6.56–7.35] vs. survival: 7.33 [7.06–7.6]; *p* < 0.05) (Table 1).

**Table 1:** Demographic and clinical characteristics of patients with pediatric myocarditis.

Characteristic	Survival (*n* = 14)	Mortality (*n* = 7)	*p* value
Demographics			
Male, n (%)	4 (28.6)	3 (42.9)	0.638
Age (month)	113 (2–167)	150 (14–180)	0.636
Height (cm)	133 (65–156)	150 (73–152)	0.911
Body weight (kg)	23 (5.6–52)	40 (8.6–50)	0.681
Body surface area (Haycock)	1.19 (0.45–1.76)	1.55 (0.57–1.71)	0.743
Days from onset to admission (day)	3 (2–10)	2 (2–4)	0.129
**Laboratory test results**			
pH	7.33 (7.06–7.6)	7.02 (6.56–7.35)	*
Lactate (mmol/L)	5.4 (1.97–16.1)	14 (1.64–16.6)	0.172
BNP (pg/mL)	658 (283–8412)	1160 (552–3849)	0.101
Troponin T (ng/mL)	1.96 (0.33–9.35)	2.72 (0–24.7)	0.971
CK-MB (IU/I)	122 (29–1580)	1832 (6–4578)	0.224
Platelet count (×10⁴/µL)	23.9 (15.3–30.8)	6.5 (3.1–40.7)	*
LVEF (%)	30.7 (9–72)	10.7 (8–40)	0.117
VT, n (%)	4 (28.6)	3 (42.9)	0.638
CAVB, n (%)	5 (35.7)	1 (14.3)	0.613

Data are presented as numbers (percentages) or medians (minimum-maximum). Body surface area was calculated using the Haycock formula. Left ventricular ejection fraction (LVEF) is reported as the percentage (%) of blood ejected from the left ventricle per heartbeat. * Indicates *p* < 0.05.

BNP, B-type natriuretic peptide; CAVB, complete atrioventricular block; CK-MB, creatine kinase-MB; LVEF, left ventricular ejection fraction; VT, ventricular tachycardia.

Furthermore, after FDR adjustment, seven cytokines demonstrated statistically significant differences between the survival and mortality groups (q < 0.05). These included TNF-α (survival: 31.5 pg/mL [3.05–75.7] vs. mortality: 91.5 pg/mL [37.0–168], *p* < 0.01, q < 0.05), M-CSF (survival: 170 pg/mL [0–1,246] vs. mortality: 1787 pg/mL [254–3625], *p* < 0.01, q < 0.05), MIP-1α (survival: 23.8 pg/mL [9.82–53.5] vs. mortality: 52.1 pg/mL [31.2–64.8], *p* < 0.01, q < 0.05), IL-8 (survival: 26.8 pg/mL [8.69–197] vs. mortality: 372 pg/mL [50.6–1,363], *p* < 0.01, q < 0.05), IL-6 (survival: 22.5 pg/mL [0.24–579] vs. mortality: 1,862 pg/mL [32.7–10,404], *p* < 0.01, q < 0.05), IL-15 (survival: 10.8 pg/mL [3.29–69.9] vs. mortality: 31.2 pg/mL [18.2–85.0], *p* < 0.01, q < 0.05), and IP-10 (survival: 683 pg/mL [117–11,374] vs. mortality: 21,636 pg/mL [1269–9,536,904], *p* < 0.01, q < 0.05) (Table 2).

**Table 2:** Comparison of cytokine levels in survival and mortality groups.

Cytokine	Survival	Mortality	*p*-value	q-value	VIP (Mean)
TNF-*α*	31.5 (3.05–75.7)	91.5 (37–168)	< 0.01	*	1.754
MIP-1*α*	23.8 (9.82–53.5)	52.1 (31.2–64.8)	< 0.01	*	1.592
M-CSF	170 (0–1246)	1787 (254–3625)	< 0.01	*	1.525
IL-8	26.8 (8.69–197)	372 (50.6–1363)	< 0.01	*	1.503
MIG	8724 (937–67021)	60,081 (8282–113605)	< 0.05	0.074	1.382
IL-6	22.5 (0.24–579)	1862 (32.7–10404)	< 0.01	*	1.312
PDGF-AB/BB	23,388 (1425–34467)	6660 (173–21229)	< 0.05	0.074	1.282
IL-22	24.3 (5.91–69)	78.6 (10.8–132)	< 0.05	0.113	1.28
MCP-1	453 (156–929)	733 (401–5089)	< 0.05	0.107	1.276
IL-15	10.8 (3.29–69.9)	31.2 (18.2–85)	< 0.01	*	1.195
IFN-α2	29.4 (7.77–92.37)	54.44 (33.51–141)	< 0.05	0.078	1.186
IP-10	683 (117–11374)	21,636 (1269–9536904)	< 0.01	*	1.11
PDGF-AA	3394 (582–5484)	1068 (448–4360)	< 0.05	0.119	1.033
IL-18	209 (44.6–1140)	403 (272–1953)	< 0.05	0.114	1.016
IL-1*α*	9.06 (0–118)	49.1 (10.1–127)	< 0.05	0.076	1.011
IFN-γ	3.47 (0.71–15.34)	6.88 (1.67–15.6)	< 0.05	0.089	0.955
G-CSF	117 (40.7–1522)	743 (96.2–10972)	< 0.05	0.086	0.952
IL-13	34 (0–69.6)	49 (35.9–122)	0.155	0.278	0.93
MIP-1β	45 (9.54–151)	73.56 (28.4–174)	0.172	0.278	0.89
IL-12p70	2.41 (0.33–6.59)	3.97 (1.28–8.31)	0.079	0.191	0.82
VEGF-A	21.25 (5.78–430)	11.08 (5.99–19.29)	0.086	0.215	0.725
IL-12p40	105 (26.64–251)	97.38 (23.1–607)	0.636	0.705	0.684
IL-10	165 (8.82–1191)	297 (123–5577)	0.197	0.352	0.68
IL-7	13.7 (0.44–33.6)	8.59 (1.56–19.33)	0.433	0.581	0.616
IL-5	2.48 (1.04–9.69)	2.48 (1.04–9.69)	0.262	0.382	0.605
IL-17 F	25.13 (3.0–225)	15.68 (3.78–240)	0.628	0.705	0.578
IL-1RA	114 (4.23–2296)	63.94 (8.15–991)	0.636	0.705	0.541
IL17E/IL-25	492 (150–1966)	909 (548–1391)	0.125	0.253	0.524
IL-1β	5.62 (1.39–59.53)	10.22 (2.85–40.26)	0.167	0.278	0.504
Eotaxin	64.42 (20.41–115)	47.0 (26.76–109)	0.535	0.688	0.503
IL-2	0.83 (0.07–6.2)	1.29 (0.07–3.38)	0.134	0.253	0.497
TNF-β	0 (0–3.87)	0.387 (0–21.2)	< 0.05	0.126	0.463
EGF	29.18 (3.19–127)	32.98 (13.95–112)	0.601	0.705	0.437
IL-4	4.74 (1.75–6.76)	4.32 (2.42–6.35)	0.681	0.724	0.401
IL-17 A	5.89 (0.77–47.11)	5.16 (0.77–22.8)	0.43	0.581	0.4
IL-3	0 (0–0.07)	0 (0–2.75)	0.174	0.278	0.278

Data are presented as numbers (percentages) or medians (minimum-maximum). Cytokine concentrations are reported in picograms per milliliter (pg/mL). q-values represent FDR-adjusted significance levels. * indicates q < 0.05. The mean VIP represents the average of the VIP score distribution obtained from 2,000 bootstrap resamples.

### Hierarchical clustering of clinical parameters and cytokines

**Fig. 2:**
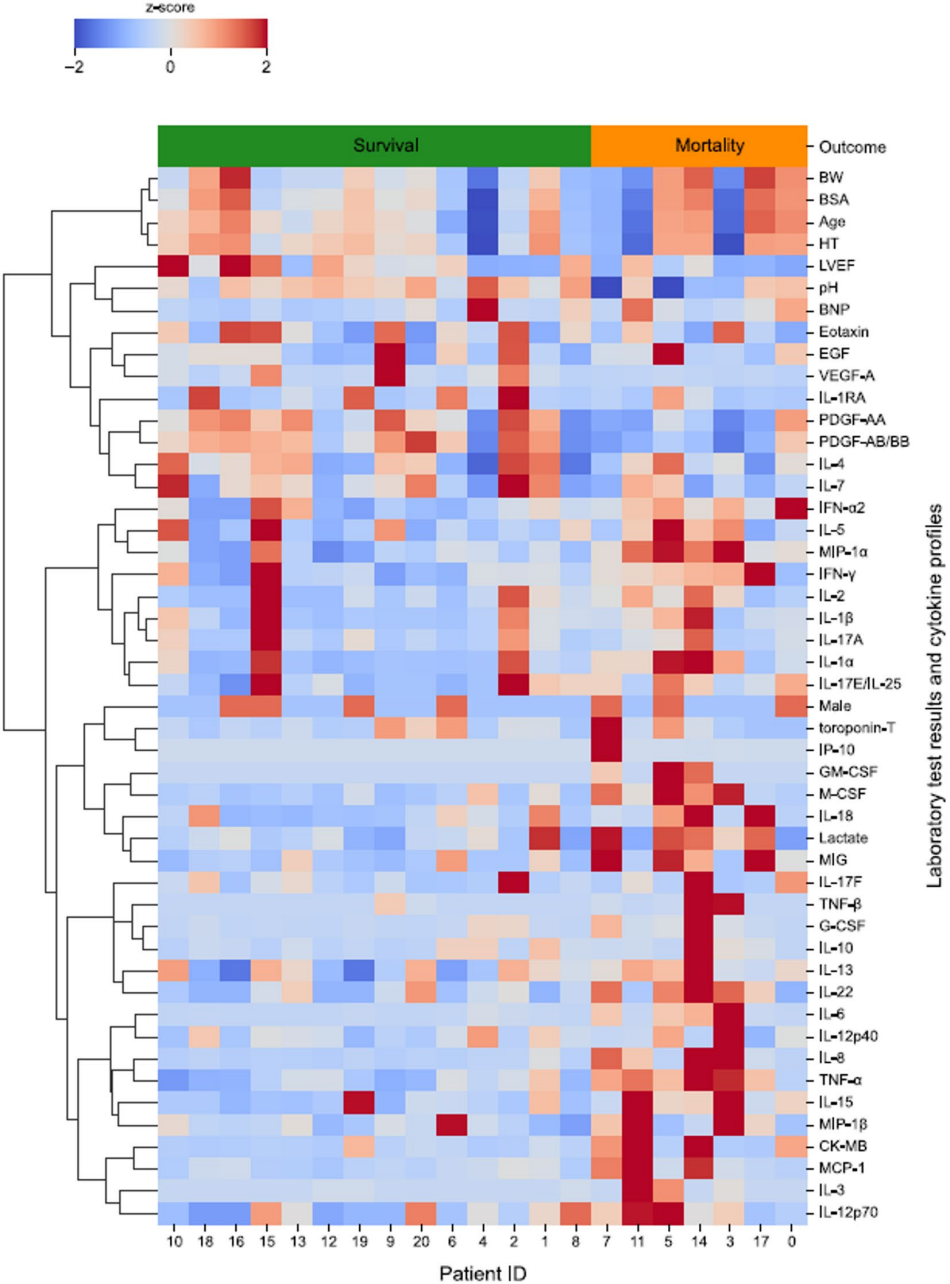
Hierarchical clustering heatmap. The top dendrogram illustrates the clustering of patients, while the side dendrogram displays the clustering of clinical characteristics and cytokines. The right side indicates the two groups (survival and mortality) analyzed. In the heatmap, each column represents an individual patient (color-coded by outcome: green for survival, orange for mortality), and each row represents a distinct clinical characteristic or cytokine, organized by expression similarity. Heatmap colors indicate cytokine expression intensities. BW, body weight; BSA, body surface area; HT, height; LVEF, left ventricular ejection fraction; BNP, B-type natriuretic peptide; CK-MB, creatine kinase-MB.

Further analysis using hierarchical clustering of clinical parameters and cytokines revealed distinct expression patterns between the survival and mortality groups (Fig. 2). The accompanying dendrograms provided additional insight into relationships among cytokines and patient profiles. While most demographic and clinical parameters showed no statistically significant group differences, clustering of cytokine profiles suggested that certain patterns, particularly among variables clustered below Male, may reflect group-specific trends. Although these clusters did not clearly segregate the survival and mortality groups, they indicate latent multivariate differences that merit further investigation. To identify more precisely key predictive features, a machine learning approach (PLS-DA) was applied for quantitative validation of observed group differences.

### PLS-DA in survival and mortality groups

The PLS-DA model addressed multicollinearity among 51 features: 14 clinical variables (six demographic parameters and eight laboratory test results) and 37 cytokines.

**Fig. 3:**
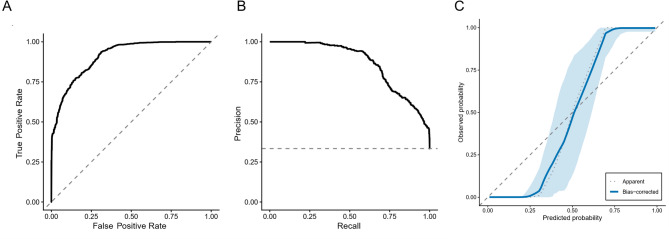
Classification performance of the evaluated model assessed by repeated 3-fold cross-validation with 100 repeats. (**A**) Receiver operating characteristic (ROC) curve obtained by averaging the ROC curves computed from fold-averaged predictions across 100 repeats of 3-fold cross-validation. The dashed diagonal line indicates chance-level discrimination. (**B**) Precision–recall (PR) curve obtained by averaging the PR curves computed from fold-averaged predictions across 100 repeats of 3-fold cross-validation. The dashed horizontal line denotes the baseline precision corresponding to the prevalence of the positive class (0.33). (**C**) Calibration plot based on bootstrap resampling, showing the apparent and bias-corrected calibration curves. The diagonal line represents perfect calibration.

Model performance was evaluated using repeated stratified 3-fold cross-validation with 100 repeats, preserving class proportions. For each repeat, predictions obtained from the three folds were averaged at the sample level, and performance metrics were computed from these fold-averaged predictions. The final performance is reported as the mean (standard deviation) across the 100 repeats. The overall accuracy was 0.838 (SD, 0.043), sensitivity was 0.710 (SD, 0.052), specificity was 0.901 (SD, 0.060), and the F1 score was 0.746 (SD, 0.057). ROC analysis showed an AUROC of 0.912 (SD, 0.032), and the AUPRC was 0.874 (SD, 0.045) (Fig. 3A, B). Furthermore, calibration analysis using bootstrap resampling demonstrated that the apparent calibration curve showed slight optimism, whereas the bias-corrected curve was closer to the ideal diagonal line (Fig. 3C). The Brier score was 0.126 (apparent) and 0.147 (bias-corrected), indicating a modest degree of optimism and overall acceptable predictive performance after correction. This suggests that, although some degree of overfitting is present, the model exhibits reasonable calibration after adjustment. However, deviations observed in the higher probability range indicate that calibration may be less reliable in extreme risk predictions. To further assess whether model performance exceeded that expected by chance, we performed a permutation test with 1,000 label shuffles. Consequently, accuracy, AUROC, and AUPRC were all significantly higher than the corresponding null distributions (Supplementary Fig. 1).

**Fig. 4:**
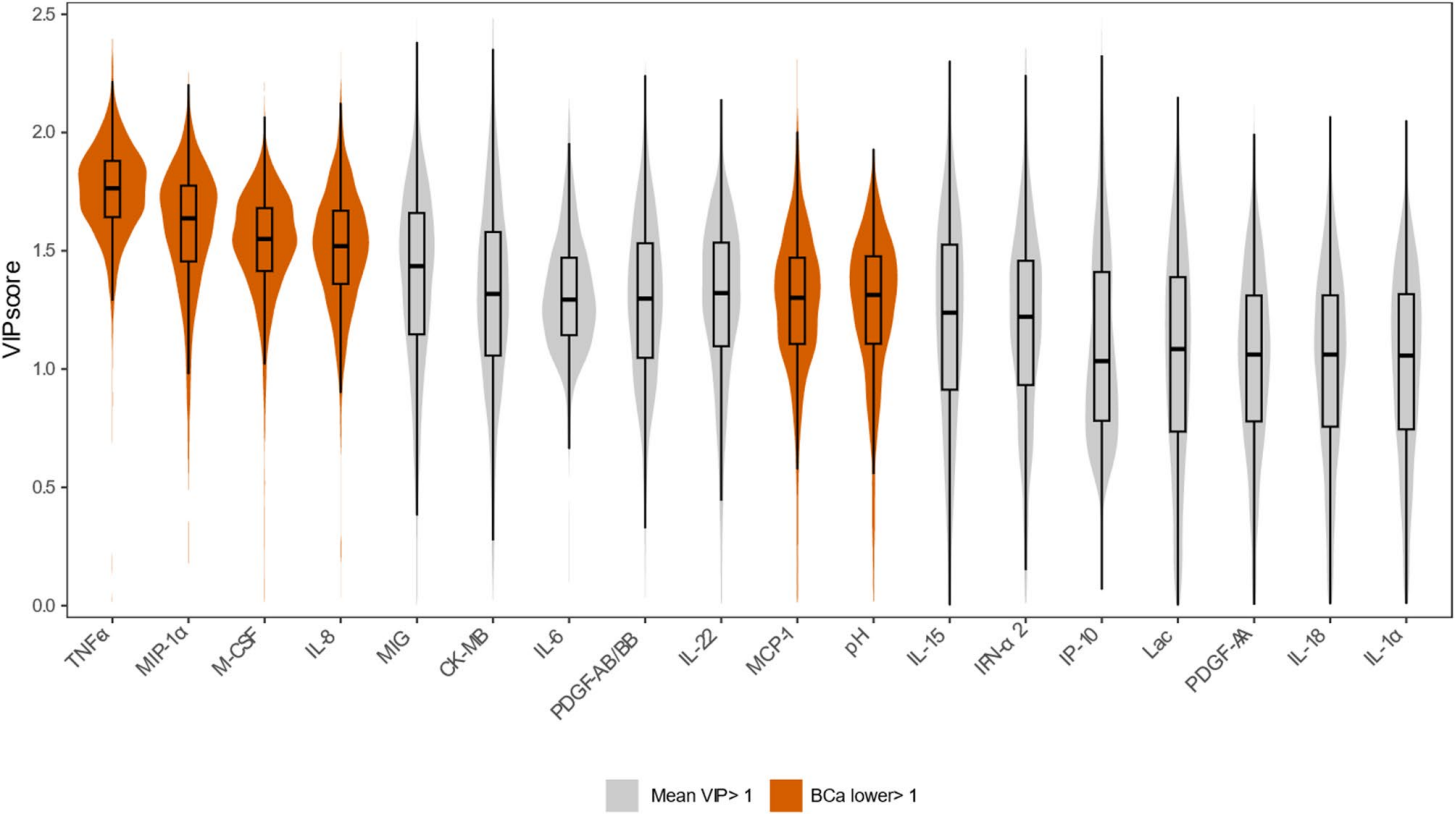
Bootstrap distributions of variable importance in projection (VIP) scores derived from PLS discriminant analysis (PLS-DA). Violin plots show the distribution of VIP scores across 2,000 bootstrap resamples for each variable, with embedded boxplots indicating the median and interquartile range. Variables are highlighted in orange when the lower bound of the bias-corrected and accelerated (BCa) bootstrap confidence interval exceeds 1, indicating stable importance across resamples, whereas variables shown in grey satisfy only the criterion of mean VIP > 1. CK-MB, creatine kinase-MB; Lac, lactate.

To identify prognostic factors, we performed 2000 bootstrap resamples for each variable in the PLS-DA model and calculated the distributions of VIP scores (Fig. 4). Confidence intervals were estimated using the bias-corrected and accelerated (BCa) bootstrap method. Variables whose lower bound of the confidence interval (lower VIP score) exceeded 1 were highlighted in orange as factors showing consistently high importance across resampling. Conversely, variables with a mean VIP score ≥ 1 but with a confidence-interval lower bound not exceeding 1 were shown in gray. A total of 18 prognostic features with mean VIP scores greater than 1.0 were identified, indicating significant contributions to mortality prediction in pediatric FM. Among all predictors, TNF-α exhibited the highest VIP score. These 18 features included three clinical laboratory variables—pH (*p* < 0.05, mean VIP = 1.261), lactate (*p* = 0.172, mean VIP = 1.046), and CK-MB (*p* = 0.224, mean VIP = 1.314)—as well as 15 of the 37 cytokines measured. Furthermore, cytokine profiling identified seven “dual-significance” biomarkers that satisfied the PLS-DA importance criterion (mean VIP > 1.0) and statistical significance after FDR correction (q < 0.05): TNF-α (mean VIP = 1.754), MIP-1α (mean VIP = 1.592), M-CSF (mean VIP = 1.525), IL-8 (mean VIP = 1.503), IL-6 (mean VIP = 1.312), IL-15 (mean VIP = 1.195), and IP-10 (mean VIP = 1.11). Conversely, six cytokines showed high importance in the PLS-DA model (mean VIP > 1.0) but did not reach statistical significance in FDR-adjusted univariate analyses (Table 2), namely IL-22, MCP-1, IFN-α2, PDGF-AA, IL-18, and IL-1α.

### Comparison of clinical-only and cytokine-enriched models

**Fig. 5:**
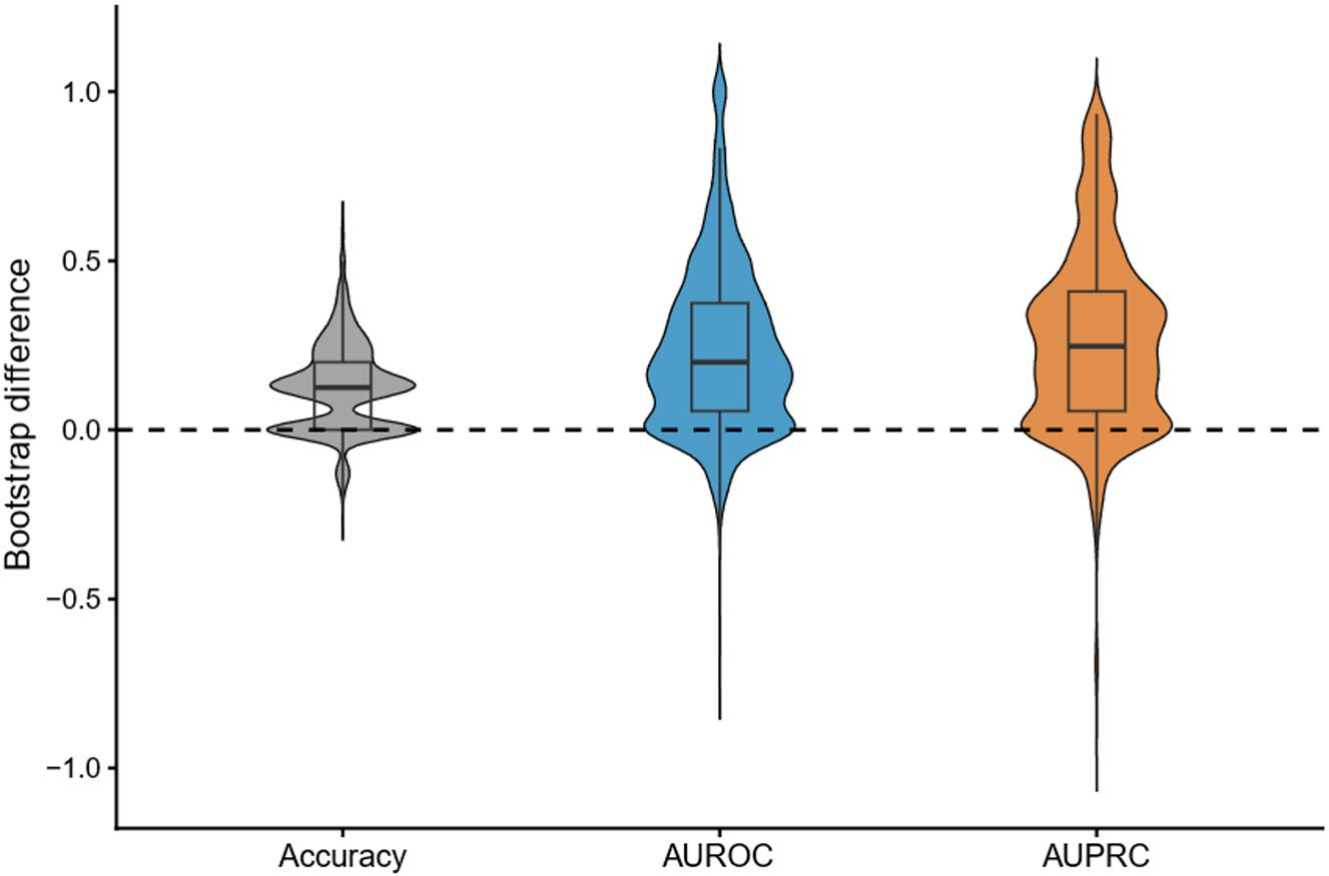
Violin plots showing paired bootstrap distributions of performance differences between models, evaluated using out-of-bag predictions. For each bootstrap resample, performance metrics were computed separately for each model, and pairwise differences were calculated as Model A minus Model B. Figure shows differences between the combined model and the clinical-only model (All − Clinical). Within each panel, differences in accuracy, AUROC, and AUPRC are shown. Boxplots indicate the median and interquartile range, and the dashed horizontal line denotes zero difference.

Finally, to evaluate the incremental value of cytokine markers, we compared the predictive performance of a clinical-only model (pH, lactate, CK-MB, ±LVEF) with that of a full model integrating clinical and cytokine variables. Performance was assessed using accuracy, AUROC, and AUPRC. To rigorously evaluate differences between the two models, we performed a paired bootstrap analysis. For each bootstrap resample, performance metrics for both models were computed from out-of-bag predictions, and the differences between the full and clinical models (full − clinical) were calculated. The distributions of differences in accuracy, AUROC, and AUPRC were visualized using violin plots (Fig. 5). The median of the difference distributions was positive for all performance metrics, indicating a consistent improvement in predictive performance with the inclusion of cytokine markers. These results suggest that cytokine markers provide additional prognostic information beyond routine clinical variables.

## Discussions

In this study, a machine learning model was applied to analyze cytokine profiles and clinical features associated with mortality in FM. The results indicated that several cytokines, including TNF-α, along with clinical parameters such as pH, lactate, and CK-MB, were significantly associated with mortality. Notably, a cytokine-enriched model consistently outperformed the clinical-only model, demonstrating that cytokine markers provide additional prognostic value beyond routine clinical variables.

Our analysis of cytokine profiles in FM identified elevated levels of IL-6, IL-8, and IL-15, and reduced levels of PDGF-AA as being associated with mortality. These cytokines were also previously examined in our study comparing AM and FM^7^. In that study, IL-8, IL-15, and PDGF-AA showed statistically significant differences between the AM and FM groups, and were identified as key cytokines distinguishing the two groups in principal component analysis. The present study suggests that these cytokines may also be linked to mortality in FM, indicating that markers of disease severity in AM may overlap with those associated with poor outcomes in FM.

TNF-α had the highest VIP score and was identified as a key cytokine for predicting mortality in patients with FM. Notably, TNF-α was not the primary focus of our previous cytokine studies aimed at stratifying AM severity^7^. Although prior studies have linked TNF-α to FM pathophysiology, paradoxically, TNF-α suppression has been reported to delay healing and worsen disease outcomes^16^. However, recent animal model studies on FM have highlighted the critical role of TNF-α in the early inflammatory response, showing that the timing of TNF-α inhibition is pivotal. Specifically, targeted suppression of TNF-α at appropriate disease stages has demonstrated improved outcomes^17^. Steroids and intravenous immunoglobulin are used in the treatment of FM. However, to the best of our knowledge, no studies have investigated their effects on cytokine responses. Longitudinal measurement of cytokine levels may provide evidence of the therapeutic efficacy of these interventions in future studies. Currently, treatment strategies for FM do not include infliximab, an anti-TNF-α agent^1^. Given the severe circulatory failure requiring MCS observed in early FM cases, anti-TNF-α therapy may warrant further investigation in FM. However, further clinical investigations in humans are needed to validate this hypothesis and explore the therapeutic role of targeted TNF-α inhibition in FM. However, a randomized controlled trial to assess anti-TNF-α therapy in FM is unlikely to be feasible due to the condition’s rarity and severity.

VIP score analysis revealed that troponin T was not significantly associated with mortality, whereas CK-MB demonstrated a significant correlation with fatal outcomes. Previous studies have similarly reported that troponin is not a risk factor for cardiac arrest or the need for MCS in pediatric myocarditis^4^. The development of FM is characterized by a rapid transition from initial viral illness-like symptoms to circulatory collapse, often resulting in fatal outcomes without timely therapeutic intervention^18^. Notably, the time from symptom onset to hospitalization is approximately 3 days^19^, a finding consistent with our current analysis. This supports the notion that FM follows a predictable progression. Regarding the kinetics of myocardial injury markers, high-sensitivity troponin rises earlier, followed by CK-MB. Troponin levels typically normalize within 7–10 days. Due to its shorter half-life, CK-MB reaches peak levels concurrent with the onset of acute circulatory failure^20^. These findings suggest that CK-MB may be a sensitive biomarker for predicting early mortality in FM. Moreover, a 2024 study using myocardial cells from FM patients demonstrated that CK-MB showed a stronger correlation with the severity of acute myocardial injury than did troponin^21^. This provides strong supporting evidence for our findings, reinforcing the prognostic utility of CK-MB in FM.

LVEF, an echocardiographic parameter reflecting systolic function, was not associated with mortality in the VIP score analysis. In contrast, the study by Zhao et al. identified LVEF as a prognostic predictor in FM^3^. However, direct comparisons between studies are limited by differences in diagnostic criteria and the accuracy of FM diagnosis. In a study on cardiogenic shock in adults, no statistically significant difference in LVEF was observed between individuals with and without cardiac arrest^22^. Similarly, LVEF may not be a reliable prognostic marker in FM, as previously reported in pediatric myocarditis, where it was not associated with cardiac arrest or the need for MCS^4^. LVEF is known to be highly variable due to its sensitivity to preload and afterload conditions, making accurate assessment difficult in the context of cardiogenic shock and high-dose catecholamine therapy. In our study, in addition to cases with irreversible myocardial damage and severe systolic dysfunction requiring ECMO support, fatal arrhythmia such as CAVB also contributed to the ECMO indication. Therefore, factors beyond systolic function may have contributed to circulatory failure, potentially influencing our results. Further investigation is warranted to clarify the prognostic significance of LVEF in FM.

In this study, although lactate levels did not show a statistically significant difference between the survival and mortality groups, they were identified as a prognostic feature with a high VIP score in the machine learning model. Clinically, lactate is widely used as a key indicator of hemodynamic status, similar to pH, but elevated lactate may be a “too late” marker for poor outcome, reflecting advanced circulatory failure^3^. These findings suggest that parameters emphasized by clinicians through experience or intuition may not reach significance in univariate analyses but can be effectively captured and weighted in machine learning models. Integrating clinical insights with objective machine learning analysis allows for the development of predictive models that are more relevant in real-world settings. Ongoing collaboration between clinical experts and data scientists will be essential in future research to identify clinically meaningful predictors and enhance the validity and utility of prognostic models.

The role of IP-10 has been previously implicated in the pathogenesis of myocarditis^23–25^, and murine model studies have shown that suppression of endogenous IP-10 leads to reductions in serum CK-MB levels and improved survival outcomes^26^. These findings suggest that IP-10 may be involved in inflammatory pathways associated with disease severity in myocarditis. Moving forward, the development of more accurate prognostic models will require refinement of machine learning approaches and expanded collection of serum cytokine profiles, particularly in individuals with FM.

In contrast to TNF-α, which was markedly elevated in mortality cases, PDGF-AB/BB concentrations were higher in survivors. Given that PDGF is partially released from activated platelets, we compared platelet counts between groups and found that platelets were markedly lower in non-survivors. This finding is consistent with the lower PDGF-AB/BB levels observed in non-survivors and suggests that PDGF reflects the degree of thrombocytopenia rather than mortality-associated inflammation. Prior studies in severe sepsis have also reported reduced PDGF-BB levels in non-survivors^27^, which parallels our observation.

Although this study advances our understanding of the application of machine learning methods to mortality prediction in pediatric myocarditis, it has certain limitations. First, this study employed a retrospective design, which introduces inherent constraints in data collection and interpretation. Retrospective studies are prone to inconsistencies in data completeness, as clinical records may not have been collected uniformly. Moreover, therapeutic interventions and disease trajectories cannot be evaluated in real time, necessitating cautious interpretation of causal relationships compared to prospective approaches. While individual variables may not have achieved statistical significance due to the limited sample size, integrated analysis using machine learning suggested a potential to discriminate between groups. Second, an imbalance in the dataset posed a key limitation. The significantly higher number of survivors than non-survivors may have reduced recall and sensitivity, particularly in mortality prediction. The small number of non-survivor cases may have limited the model’s capacity to learn discriminative patterns, potentially resulting in an underestimation of mortality risk. However, pediatric FM is an extremely rare condition, and data collection is inherently challenging due to its low prevalence and the need for specialized clinical settings. Third, although the exact timing of FM onset remains uncertain in many cases, variations in disease progression are likely minimal, as the condition generally follows a consistent clinical course. Therefore, discrepancies in the timing of cytokine and clinical marker measurement are unlikely to influence study outcomes meaningfully. Nevertheless, the precise timing of myocardial injury marker elevations (e.g., CK-MB and troponin) in early-stage FM remains unclear and should be investigated in future studies. Fourth, cytokine levels were measured only at admission. Repeated cytokine measurements are often impractical in real-world pediatric settings, and therefore, the temporal evolution of cytokine profiles during disease progression and treatment is unknown, limiting insight into their dynamic role in clinical outcomes. In addition, the small sample size relative to the number of candidate predictors may increase the risk of overfitting and unstable feature importance, despite the use of cross-validation and bootstrap-based analyses. Therefore, the present model should be interpreted as exploratory. Moreover, this study lacked external validation using an independent cohort, which limits the generalizability of our findings and warrants confirmation in larger, independent populations. Larger studies will be needed to confirm the robustness of feature selection. Finally, although TNF-α emerged as a strong predictor in our model, its direct clinical applicability as a therapeutic target in pediatric FM remains uncertain. Experimental or longitudinal studies are needed to clarify whether modulation of TNF-α has therapeutic value.

## Conclusions

This study highlights the potential of machine learning models to predict outcomes in pediatric FM by integrating clinical features and cytokine profiling. The observed prognostic associations between cytokines, particularly TNF-α, and prognosis support further investigation into the role of cytokine pathways in pediatric FM.

## Electronic Supplementary Material

Below is the link to the electronic supplementary material.

Supplementary Material 1

Supplementary Material 2

## Data Availability

The datasets and code used in this study are available at: https://github.com/sakulab-org/pediatric-myocarditis-cytokine-prediction.

## Abbreviations

AM: Acute myocarditis
AUROC: Area under the receiver operating characteristic curve
AUPRC: Area under the precision–recall curve
BNP: B-type natriuretic peptide
CAVB: Complete atrioventricular block
CK-MB: Creatine kinase MB
ECMO: Extracorporeal membrane oxygenation
FDR: False discovery rate
FM: Fulminant myocarditis
FN: False negatives
FP: False positives
LVEF: Left ventricular ejection fraction
MCS: Mechanical circulatory support
PLS-DA: Partial least square discriminant analysis
ROC: Receiver operating characteristic curve
TNF-α: Tumor necrosis factor-alpha
TN: True negatives
TP: True positives
VIP: Variable importance in projection
VT: Ventricular tachycardia

## References

[1] Kociol, R. D. et al. Recognition and initial management of fulminant myocarditis: a scientific statement from the American Heart Association. *Circulation* **141**, e69–e92 (2020). 31902242 10.1161/CIR.0000000000000745

[2] Nagai, T. et al. JCS 2023 Guideline on the diagnosis and treatment of myocarditis. *Circ. J.* **87**, 674–754 (2023). 36908170 10.1253/circj.CJ-22-0696

[3] Zhao, Y., Da, M., Yang, X., Xu, Y. & Qi, J. A retrospective analysis of clinical characteristics and outcomes of pediatric fulminant myocarditis. *BMC Pediatr.* **24**, 553 (2024). 39210278 10.1186/s12887-024-05022-4 PMC11360287

[4] Casadonte, J. R. et al. Risk factors for cardiac arrest or mechanical circulatory support in children with fulminant myocarditis. *Pediatr. Cardiol.* **38**, 128–134 (2017). 27826709 10.1007/s00246-016-1493-5

[5] Amioka, N. et al. Pathological and clinical effects of interleukin-6 on human myocarditis. *J. Cardiol.* **78**, 157–165 (2021). 33814251 10.1016/j.jjcc.2021.03.003

[6] Calabrese, F. et al. Overexpression of tumor necrosis factor (TNF)alpha and TNFalpha receptor I in human viral myocarditis: clinicopathologic correlations. *Mod. Pathol.* **17**, 1108–1118 (2004). 15218506 10.1038/modpathol.3800158

[7] Nomura, Y. et al. Analysis of cytokine profiles in pediatric myocarditis multicenter study. *Pediatr. Cardiol.* **46**, 544–552 (2025). 38480571 10.1007/s00246-024-03452-6

[8] Adel-Patient, K. et al. A comprehensive analysis of immune constituents in blood and bronchoalveolar lavage allows identification of an immune signature of severe asthma in children. *Front. Immunol.* **12**, 700521 (2021). 34349761 10.3389/fimmu.2021.700521 PMC8327906

[9] Zheng, H. H. et al. Identification of canine pyometra-associated metabolites using untargeted metabolomics. *Int. J. Mol. Sci.* **23**, 14161 (2022). 36430638 10.3390/ijms232214161 PMC9697130

[10] Oliveira, M. S. et al. Urinary metabolomic biomarker candidates for skeletal muscle wasting in patients with rheumatoid arthritis. *J. Cachexia Sarcopenia Muscle*. **14**, 1657–1669 (2023). 37243418 10.1002/jcsm.13240 PMC10401545

[11] Barker, M. & Rayens, W. Partial least squares for discrimination. *J. Chemometrics*. **17**, 166–173 (2003).

[12] Writing Committee, Drazner, M. H. et al. 2024 ACC expert consensus decision pathway on strategies and criteria for the diagnosis and management of myocarditis: a report of the American College of Cardiology Solution Set Oversight Committee. *J. Am. Coll. Cardiol.* **85**, 391–431 (2025). 39665703 10.1016/j.jacc.2024.10.080

[13] Singh, D. & Singh, B. Investigating the impact of data normalization on classification performance. *Appl. Soft Comput.* **97**, 105524 (2020).

[14] Ward, J. H. & Hook, M. E. Application of a hierarchical grouping procedure to a problem of grouping profiles. *Educ. Psychol. Meas.* **23**, 69–81 (1963).

[15] Murtagh, F. & Legendre, P. Ward’s hierarchical agglomerative clustering method: which algorithms implement Ward’s criterion? *J. Classif.* **31**, 274–295 (2014).

[16] Cautela, J. et al. Intensified immunosuppressive therapy in patients with immune checkpoint inhibitor-induced myocarditis. *J. Immunother Cancer*. **8**, e001887 (2020). 33298621 10.1136/jitc-2020-001887 PMC7725077

[17] Rolski, F. et al. TNF-α protects from exacerbated myocarditis and cardiac death by suppressing expansion of activated heart-reactive CD4 + T cells. *Cardiovasc. Res.* **120**, 82–94 (2024). 37879102 10.1093/cvr/cvad158 PMC10898940

[18] Law, Y. M. et al. Diagnosis and management of myocarditis in children: a scientific statement from the American Heart Association. *Circulation* **144**, e123–e135 (2021). 34229446 10.1161/CIR.0000000000001001

[19] Matsuura, H. et al. clinical features of acute and fulminant myocarditis in children – 2nd nationwide survey by the Japanese Society of Pediatric Cardiology and Cardiac Surgery. *Circ. J.* **80**, 2362–2368 (2016). 27725476 10.1253/circj.CJ-16-0234

[20] Nagajothi, N. & Trivedi, A. Biomarkers in acute cardiac disease. *J. Am. Coll. Cardiol.* **48**, 2358 (2006). author reply 2358–2359. 17161280 10.1016/j.jacc.2006.10.013

[21] Toyoda, S. et al. Accumulation of endogenous muse cells in the myocardium and its pathophysiological role in patients with fulminant myocarditis. *Clin. Transl Sci.* **17**, e70067 (2024). 39543854 10.1111/cts.70067 PMC11564339

[22] Tabi, M. et al. Echocardiographic characteristics of cardiogenic shock patients with and without cardiac arrest. *J. Intensive Care Med.* **38**, 51–59 (2023). 35656768 10.1177/08850666221105236

[23] Fujii, M. et al. Multidisciplinary diagnostic approach for fulminant myocarditis related to coronavirus disease 2019 messenger RNA vaccines: a case report. *Eur. Heart J. Case Rep.* **7**, ytad063 (2023). 36819885 10.1093/ehjcr/ytad063 PMC9933941

[24] Ma, P. et al. Expansion of pathogenic cardiac macrophages in immune checkpoint inhibitor myocarditis. *Circulation* **149**, 48–66 (2024). 37746718 10.1161/CIRCULATIONAHA.122.062551 PMC11323830

[25] Huang, Y. V. et al. Novel therapeutic approach targeting CXCR3 to treat immunotherapy myocarditis. *Circ. Res.* **136**, 473–490 (2025). 39931812 10.1161/CIRCRESAHA.124.325652 PMC11867805

[26] Yue, Y., Gui, J., Ai, W., Xu, W. & Xiong, S. Direct gene transfer with IP-10 Mutant ameliorates mouse CVB3-induced myocarditis by blunting Th1 immune responses. *PLoS One*. **6**, e18186 (2011). 21445362 10.1371/journal.pone.0018186 PMC3062568

[27] Brueckmann, M. et al. Prognostic value of platelet-derived growth factor in patients with severe sepsis. *Growth Factors*. **25** (1), 15–24 (2007). 17454146 10.1080/08977190701272784

