# Supplementary material for: Machine learning-based mortality prediction for pediatric fulminant myocarditis using cytokine profiles
Source: Sci Rep. 2026 Apr 24;16:22470. doi: 10.1038/s41598-026-50260-4 (PMC13376642; doi:10.1038/s41598-026-50260-4)
Supplement: Supplementary file 2 — Supplementary Material 2 [file 41598_2026_50260_MOESM2_ESM.docx]

**Supplementary Fig. 1**: Using a permutation test with random label shuffling, we evaluated whether model performance exceeded chance level. Under the null distribution, (A) mean accuracy, (B) AUROC, and (C) AUPRC were 0.59 (SD = 0.08), 0.53 (SD = 0.13), and 0.41 (SD = 0.12), respectively. The observed performance was significantly higher than the null distribution for all metrics (empirical p-values ≤ 0.003).

**
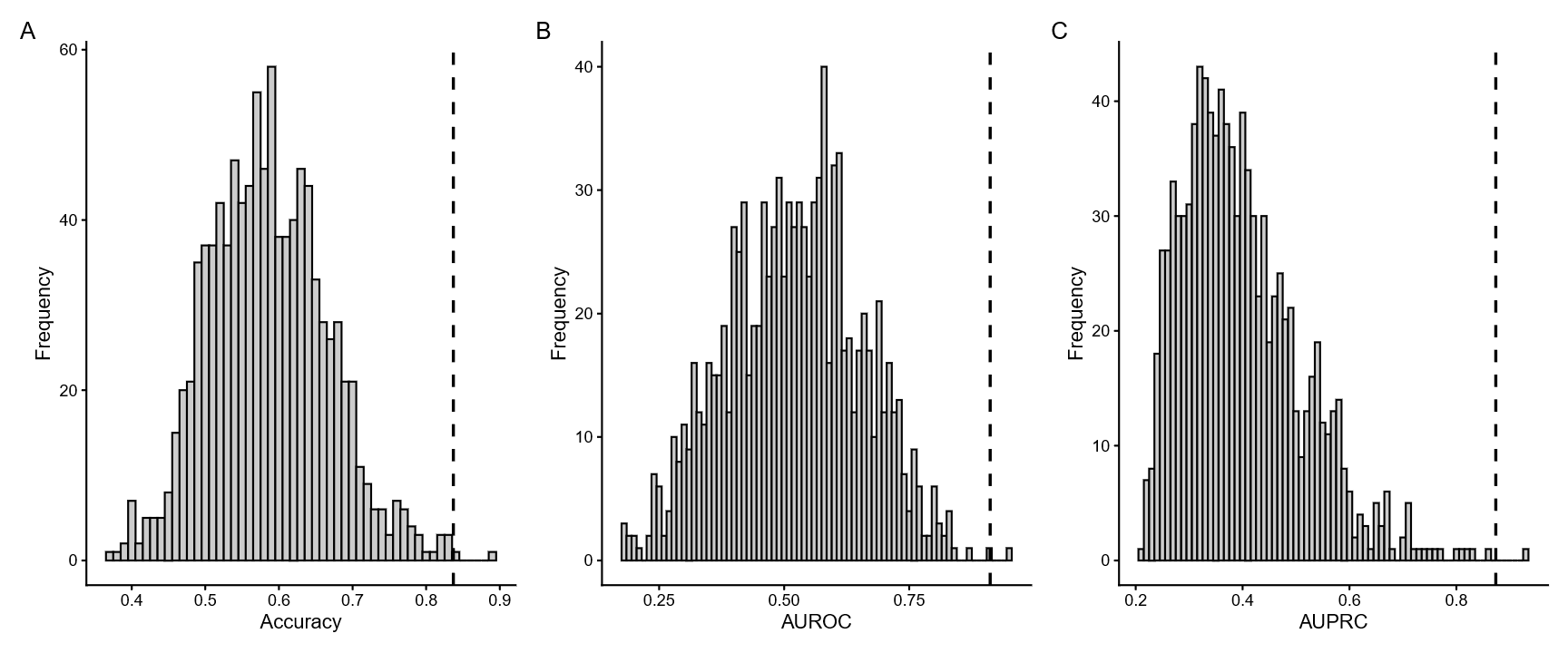
**
